# Patient reported experiences of CT guided lung biopsy: a prospective cohort study

**DOI:** 10.1186/2049-6958-9-53

**Published:** 2014-10-31

**Authors:** Naomi Winn, Jonathan Spratt, Enid Wright, Julie Cox

**Affiliations:** Robert Jones and Agnes Hunt Orthopaedic Hospital NHS Foundation Trust, Oswestry, Shropshire, UK; Sunderland General Hospital, Kayll Road, Sunderland, UK; County Durham and Darlington NHS Foundation Trust, University Hospital North Durham, Durham, UK; Northumbria Healthcare Trust, Hexham General Hospital, Corbridge Rd, Hexham Northumberland, UK

**Keywords:** CT guided biopsy, Lung carcinoma

## Abstract

**Background:**

CT guided lung biopsy is a commonly performed procedure to obtain tissue for a histological diagnosis in cases of suspected lung cancer.

**Methods:**

This is a prospective cohort study to obtain information directly from patients about their experiences of the biopsy procedure, thus obtaining a more accurate picture of complications compared with previously performed retrospective reviews. Patients participated in a post-procedure telephone interview and information was gathered about any procedural complications and personal experiences. We also compared the patient reported complications with those obtained from a retrospective review of hospital databases, analogous to previously performed retrospective studies.

**Results:**

In our patient group, reported procedural complication rates were 10% pneumothorax rate (4% requiring a chest drain) and 10% haemoptysis. Post-procedural pain and shortness of breath showed positive correlation, with one patient experiencing prolonged pain. No statistical difference was found between the patient reported complication rates and those obtained from retrospective review of the hospital database.

**Conclusions:**

Our study demonstrates CT guided lung biopsy is a safe procedure and is generally well tolerated. Some patients may experience significant and lasting pain and therefore should be counselled about this pre-procedure.

## Background

CT guided lung biopsy is a common radiological procedure performed throughout the world. It is frequently used to obtain tissue to confirm a suspected diagnosis of lung cancer to enable the histological analysis necessary for treatment planning. The procedure may also be performed for non-malignant disease, such as to obtain a specimen for microbial analysis in suspected infection. However, the majority of biopsies are for malignant disease.

Within the County Durham and Darlington Foundation Trust approximately 120 CT guided lung biopsies are performed per year across the three hospital sites. Complications of CT guided lung biopsy have been well documented and include pneumothorax (4-60%), pneumothorax requiring chest drain (5-10%), haemoptysis (10%), pain, air embolism, atrial fibrillation, tumour seeding of the biopsy tract and, on rare occasions, death (0.5%) [1–13]. To our knowledge and after an extensive literature search, all studies and procedure audits performed to date have been retrospective analyses of hospital databases, PACS (Picture Archiving and Communication System) image databases and patient notes. There have been no prospective studies directly questioning patients regarding their experiences. We propose that the reported complication rates are under-representative and only include those patients who presented to the specific hospital in the study. Patients who represented post lung biopsy to a different hospital, who consulted or sought advice from primary care services or who had symptoms after the biopsy but did not seek medical treatment would be missed from the current literature. Any less severe complications (such as minor haemoptysis) not requiring physician input would also be missed. We believe that by consulting the patients directly a more accurate assessment of the true complication rates of CT guided lung biopsy will be possible.

### Research questions

#### Primary objective

What is the patient reported complication rate following CT guided lung-biopsy?

#### Secondary objective

Are patients adequately informed regarding the procedure and aftercare?

## Methods

This was a prospective cross sectional survey of 49 patients (40% recruitment rate) who underwent CT guided lung biopsy within County Durham and Darlington Foundation Trust. Ethical approval was granted by the County Durham and Tees Valley Ethics Committee and funding was provided by County Durham and Darlington Foundation Trust.

Adult patients with capacity were eligible for inclusion in the study if they had previously undergone a CT examination of the chest demonstrating an abnormality within the chest that was suspected to be malignant or indeterminate. Patients were excluded from the study if they were under 18 years of age, lacked capacity or were not contactable by telephone. Patients were invited to participate in the study during a clinic appointment with the referring physician and an information leaflet was supplied. Further information about the study was given by the research nurse prior to the procedure and written and informed consent was taken to participate in the study. The patients were re-assured that they could withdraw their consent at any time without detriment to their future care. Consent for the procedure was taken by the physician performing the biopsy, separately from the consent for involvement in the study.

The CT guided biopsy was performed to a standard technique by one of two Consultant Radiologists (JS, PO): the patient was placed within the CT scanner and a suitable position for biopsy was ascertained. Local anaesthetic was infiltrated into the sub-cutaneous tissues and an 18G core biopsy needle was placed within the lesion. Usually, one to three samples were taken, depending on radiologist preference and the subjective opinion of whether an adequate sample had been obtained. No pathology service was available to analyse the sample at the time of the biopsy to check for adequacy. Routine post-procedure care included observation within the hospital for four hours. Additionally, a post-procedure chest radiograph was obtained to determine whether the patient had sustained a pneumothorax. Patients who were well post biopsy were discharged home after four hours. If the patient was unwell, then local hospital admission was arranged.

Within two weeks following the procedure, the patients were telephoned by the study nurse and a structured telephone interview was undertaken. It was made clear to the patients at the time of the biopsy that the purpose of the telephone interview was to discuss the biopsy procedure and not the biopsy results. The patients were informed that the biopsy results would be obtained from the referring physician during their next clinic appointment and that the study nurse did not have any information about the biopsy results.

The questions asked during the telephone interview were standardised (Table 1).

**Table 1:** **Questions asked during telephone interview**

Procedural	Expectations
Pain (duration and score out of ten)	Was the biopsy what they expected?
Immediate shortness of breath (score out of ten)	What information were they given before the biopsy and by whom?
Haemoptysis – estimate of volume (for example tea spoon-full, egg cup-full)	Did they feel that more information is needed before arriving at the hospital for the biopsy?
Additional procedures following biopsy (for example chest drain insertion)?	How could the service be improved?
Length of stay post procedure?	
Did they need to consult primary care services – GP/NHS direct?	
Were they admitted to any other hospital since the procedure?	

On average, the interview lasted 20 minutes. All patients who agreed to participate in the study were contacted post-biopsy and no patients withdrew their consent. The qualitative information was initially recorded on paper and then collated into a database.

### Statistical analysis

Statistical tests were performed using StatsDirect (http://www.statsdirect.com). The number of patients recruited into our study gives 80% power to detect a 20% difference between the two groups. The Fisher exact test was used to compare the patient reported rates of haemoptysis and pneumothorax against the rates from retrospective review of the hospital records, with no difference between the two groups evident in the results.

Spearman rank correlation test shows a strong positive correlation between the immediate pain scores and shortness of breath scores (p = 0.0022). Positive correlation is also demonstrated between the immediate and one week pain scores (p = 0.0607).

## Results

In total, 49 patients were recruited to the study over 15 months. There were 28 male and 21 female patients, with ages ranging from 34 to 87.

### Patient reported results

**Figure 1:**
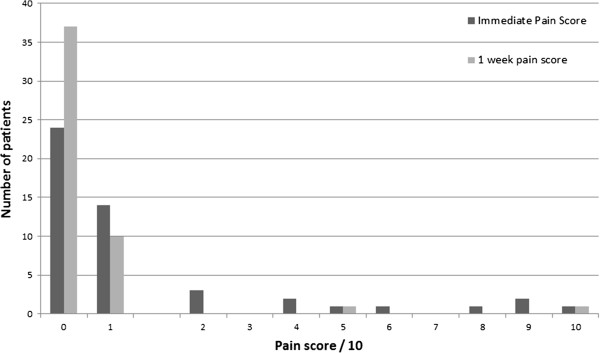
**Pain scores immediately post procedure and at one week.**

Ten out of 49 (20%) patients described the procedure as painful (Figure 1).

**Figure 2:**
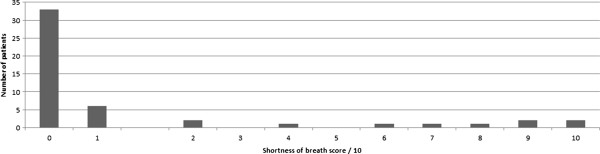
**Shortness of breath score (immediate).**

**Figure 3:**
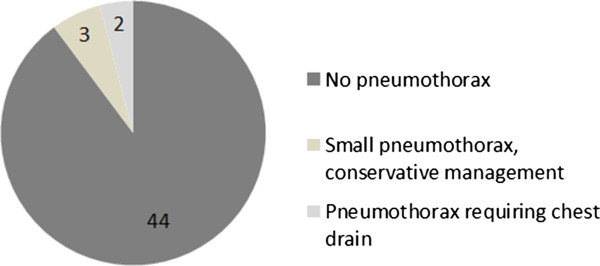
**Pneumothorax rate.**

**Figure 4:**
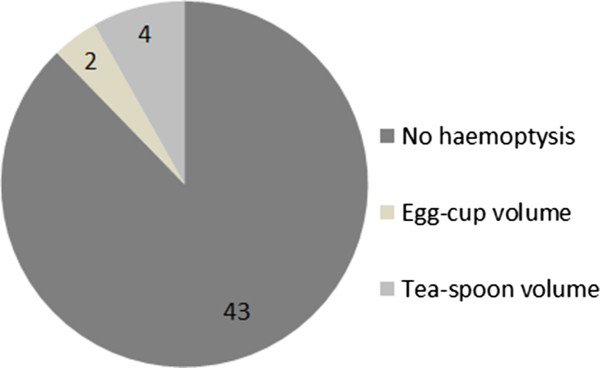
**Haemoptysis rate.**

Fourteen out of 49 (29%) patients were short of breath immediately following the procedure (Figure 2). Two out of 49 patients required an immediate chest drain (4%) (Figure 3). Six (12%) patients reported haemoptysis immediately following the procedure, of which, two (4%) patients reported haemoptysis of a teaspoon and four (8%) an egg-cup full (Figure 4).

Forty six (94%) patients were discharged on the same day of the biopsy following uneventful 4 hour post-procedure observation. One patient required an overnight stay, one patient stayed for two days and one patient required hospitalisation for more than two days. The patients were all admitted to Darlington Memorial Hospital, within County Durham and Darlington Foundation Trust.

There were no additional complications occurring between hospital discharge and the post-procedure interview at two weeks.

All forty six patients (94%) answered “yes” to the question “was the biopsy what they were expecting?” whereas 3 patients answered “no”. No patients felt that they required more information and none volunteered any additional comments.

### Retrospective review of hospital records

Review of the hospital records showed that 5 out of 49 (10%) patients sustained a pneumothorax. Two patients required a chest drain, in concordance with the patient reported rate. Out of this group, one patient sustained a large pneumothorax, requiring a chest drain and hospitalisation for greater than two days. A further patient had a 40% pneumothorax requiring a chest drain and an overnight stay in hospital. The three other patients sustained small apical pneumothoraces, none requiring a chest drain. One of these patients with a small apical pneumothorax required a two day in-patient admission.

Five patients were recorded as having post-procedural haemoptysis (10%) and 44 as none. No patients required any active treatment of the haemoptysis (such as embolisation) and all settled with conservative management.

## Discussion

### Study results

20% of our patients answered “yes” to the question “was the procedure painful?” Therefore, 80%, or the majority, tolerated it without discomfort. Similarly, 29% of our patients were short of breath during or immediately after the procedure and, therefore, 71% tolerated it without significant dyspnoea. Our results show positive correlation between immediate pain and shortness of breath, indicating that those patients who were in painwere also short of breath. This result may be expected intuitively. If the procedures were poorly tolerated, a patient may experience more pain and dyspnoea.

Of interest, in our opinion, although only 10 patients answered “yes” to the direct question about procedural pain, 25 patients scored their immediate level of pain as greater than 0 (i.e. some pain) with the majority scoring 1-2/10 for pain. This suggests that there was a group of people who experienced discomfort/low pain scores immediately following the procedure but chose not to describe the procedure overall as painful. Therefore, just under half of the patients found the procedure painful to some extent, but chose not to admit it on direct questioning. A methodological strength of our study is that it was prospective and the researchers were not part of the team providing immediate clinical care. This may have led to more honesty from the patients about the complications, particularly the peri and post procedural pain.

A positive correlation is also shown between immediate pain scores and scores at one week. This could be explained on the basis of an individual’s pain threshold. Those patients who scored their pain level as > 5 immediately, also gave relatively high scores at one week. All but one patient scored their pain level as less at one week, compared with the immediate post procedure pain scores. It is relevant that the single patient who scored his pain greater at one week compared with immediately post procedure sustained a small pneumothorax, managed conservatively.

Pain is subjective and a small number of patients found the procedure painful and experienced sustained pain for up to one week following the procedure.

Three patients said the procedure was not what they were expecting, but did not offer further comments. No patients said they required more information and none needed to consult other medical services. No patients were admitted outside our trust and thus our data reflect the true complication rates for our institution, with no late complications. No patients offered any suggestions for improvement in the service. We can surmise from the data that overall, the day-case procedure using local anaesthetic is well tolerated by our patients.

### New techniques in CT guided lung biopsy

Newer techniques in image guided biopsy offer the potential to further improve the patient experience. Cone beam CT is a relatively new technique whereby a three dimensional CT image is generated with a rotating fluoroscopic C-arm. This has the advantage of allowing an open environment, rather than an enclosed bore CT scanner. It also allows greater flexibility in the imaging planes used, as it is not limited to the range of movement of the CT gantry, with options for computer aided navigation for needle placement. Recent studies have shown similar complication rates with cone beam CT guided lung biopsy compared with conventional CT guided procedures [3, 4, 14, 15], demonstrating equivalence in complication rates. Further modifications in CT guided biopsy technique have been shown to affect the biopsy complication rate, for example CT fluoroscopy mode versus 3 slice biopsy mode and spiral acquisition versus biopsy mode. The spiral acquisition mode generally entails a longer procedure time (and increased patient dose), thus contributing to the increased complication rate during the procedure [16]. Our institution uses the 3 slice biopsy mode, minimising the procedure time and thereby reducing the risk of complications through the prolonged procedure time. The majority of the published studies to date are also from centres using the 3 slice biopsy mode, thus our practice is in concordance with other institutions [6, 8–11, 17–21].

### Complication rates from the literature

The percentage of post-procedure pneumothorax in our patient cohort (10%) is below the average value cited in the literature in similar sized studies (15%). Likewise, our percentage of pneumothorax requiring chest drain (4%) is below the values quoted in multiple studies (5-10%) [3, 8, 9, 11, 12, 17, 18, 22–25]. Our complication rates are also in accordance with a recent large retrospective populational study including > 22,000 CT guided lung biopsies, which demonstrated a pneumothorax rate of 15%, requiring a chest drain in 6.6% of all cases [1]. A study by Carlson et al. shows lower complication rates in CT guided lung biopsies performed in 2003–2005 compared with 1996–1998, implying a learning curve for the operator and possibly improvements in equipment [17]. The two Consultant Radiologists performing the lung biopsies at our institution both have at least ten year experience in performing the biopsies, with no problems encountered during any potential learning curve. The study was performed over 15 months without any equipment updates; therefore this is unlikely to be a confounding factor in our study.

Whilst our study did not take into account specific factors regarding the biopsy procedure (for example, lesion size, depth, needle gauge), we assume that our patient group is comparable to the other patient groups (non-selective inclusion) and, therefore, our results are both representative and comparable. We can be reassured that our results compare favourably with the national averages. We believe we are now able to reliably inform our patients about the risks of the procedure performed at our institutions.

Our results show that there is no difference between the patient reported complication rates for pneumothorax and haemoptysis, refuting our initial hypothesis. Thus, in our patient cohort within County Durham and Darlington Foundation Trust, the hospital recorded complication rates are an accurate assessment of the true complication rates.

Three patients required hospital admission for management of a pneumothorax immediately following the procedure (Table 2). None of our patients sustained a delayed pneumothorax. We can conclude that our practice of performing the procedure as a day-case with four hour post-procedural observation and chest radiograph is safe, with no patients sustaining a delayed serious complication. This is in accordance with guidance from the British Thoracic Society [10].

**Table 2:** **Subgroup of patients who sustained a pneumothorax**

Size of pneumothorax	Immediate pain score/10	One week pain score/10	Shortness of breath score/10	Hospital admission?	Chest drain?
Large	9	1	10	Yes	Yes
Medium (40%)	2	0	9	Yes	Yes
Small apical	6	1	8	Yes	No
Small apical	0	0	1	No	No
Small apical	1	0	0	No	No

### Limitations

One limitation of our study is the small patient cohort, which limits the power of the study. In addition, all patients are residents of County Durham and our data may not necessarily be representative of a different patient group, for example one within an urban area with a different demographic profile.

## Conclusions

Our study confirms that the described procedure is safe to perform as a day-case and tolerated well by most patients. The majority of our patients felt adequately informed about the procedure. However, an area for development is to counsel about the potential for some patients to experience more prolonged post-procedural pain, with a strategy for managing this pain.

Our complication rates are commensurate with the reported rates in the literature and there is no significant difference between the patient reported rates and those obtained from retrospective review in our patient cohort. We now know the true complication rates for our institutions and can advise our patients accordingly.
